# Role of Genetics in the Etiology of Autistic Spectrum Disorder: Towards a Hierarchical Diagnostic Strategy

**DOI:** 10.3390/ijms18030618

**Published:** 2017-03-12

**Authors:** Cyrille Robert, Laurent Pasquier, David Cohen, Mélanie Fradin, Roberto Canitano, Léna Damaj, Sylvie Odent, Sylvie Tordjman

**Affiliations:** 1 Pôle Hospitalo-Universitaire de Psychiatrie de l’Enfant et de l’Adolescent (PHUPEA), University of Rennes 1 and Centre Hospitalier Guillaume Régnier, 35200 Rennes, France; robertcyrille@live.fr; 2 Service de Génétique Clinique, Centre de Référence Maladies Rares Anomalies du Développement (Centre Labellisé pour les Anomalies du Développement de l’Ouest : CLAD Ouest), Hôpital Sud, Centre Hospitalier Universitaire de Rennes, 35200 Rennes, France; laurent.pasquier@chu-rennes.fr (L.P.); Melanie.FRADIN@chu-rennes.fr (M.F.); Lena.DAMAJ@chu-rennes.fr (L.D.); Sylvie.Odent@chu-rennes.fr (S.O.); 3 Hospital-University Department of Child and Adolescent Psychiatry, Pitié-Salpétrière Hospital, Paris 6 University, 75013 Paris, France; david.cohen@psl.aphp.fr; 4 Division of Child and Adolescent Neuropsychiatry, University Hospital of Siena, 53100 Siena, Italy; r.canitano@ao-siena.toscana.it; 5 Laboratory of Psychology of Perception, University Paris Descartes, 75270 Paris, France

**Keywords:** autism, genetic disorders, hierarchical diagnostic strategy, child psychiatric and psychological assessment, clinical genetics, neuropediatric evaluation

## Abstract

Progress in epidemiological, molecular and clinical genetics with the development of new techniques has improved knowledge on genetic syndromes associated with autism spectrum disorder (ASD). The objective of this article is to show the diversity of genetic disorders associated with ASD (based on an extensive review of single-gene disorders, copy number variants, and other chromosomal disorders), and consequently to propose a hierarchical diagnostic strategy with a stepwise evaluation, helping general practitioners/pediatricians and child psychiatrists to collaborate with geneticists and neuropediatricians, in order to search for genetic disorders associated with ASD. The first step is a clinical investigation involving: (i) a child psychiatric and psychological evaluation confirming autism diagnosis from different observational sources and assessing autism severity; (ii) a neuropediatric evaluation examining neurological symptoms and developmental milestones; and (iii) a genetic evaluation searching for dysmorphic features and malformations. The second step involves laboratory and if necessary neuroimaging and EEG studies oriented by clinical results based on clinical genetic and neuropediatric examinations. The identification of genetic disorders associated with ASD has practical implications for diagnostic strategies, early detection or prevention of co-morbidity, specific treatment and follow up, and genetic counseling.

## 1. Introduction

Autism Spectrum Disorder (ASD) is considered as a neurodevelopmental disorder defined in the DSM-5 (the Diagnostic and Statistical Manual of Mental Disorders 5th ed.) [1], the most recent diagnostic classification of mental disorders, by social communication deficits associated with repetitive/stereotyped behaviors or interests with early onset (referenced in the DSM-5 as symptoms present at early developmental period and evident in early childhood). According to the historian Normand J. Carrey, the first description of autism can be traced to Itard in his 1828 report on “mutism produced by a lesion of intellectual functions” [2]. In 1943, Leo Kanner [3], American psychiatrist with Austrian origins, borrowed the term *autism* from the Swiss psychiatrist Eugen Bleuler to define a specific syndrome observed in 11 children (the term “autism”, derived from the Greek autos which means “oneself”, was used for the first time by Eugen Bleuler in 1911 [4] to describe social withdrawal in adult patients with schizophrenia). The Kanner syndrome was characterized by its early onset (from the first year of life) and symptomatology (social withdrawal, sameness, language impairment, stereotyped motor behaviors, and intellectual disability). It is noteworthy that Kanner did not describe autism in individuals with severe intellectual disability and known brain disorders. The same year, the Austrian psychiatrist Hans Asperger [5] distinguished “personalities with autistic tendencies” which differed from the children that Kanner had described, due to the expression of exceptional isolated talents and conserved linguistic abilities. Currently, autism is viewed as an epistatic and multifactorial disorder involving genetic factors and environmental factors (prenatal, neonatal and/or postnatal environmental factors such as gestational diabetes, neonatal hypoxia conditions, exposure to air pollution, parental immigration or sensory/social deficits; for a review, see Tordjman et al., 2014 [6]).

Several literature reviews underline the important role of genetics in the etiology of autism [7,8,9]. The data come from family and twin studies. Indeed, the concordance rate among monozygotic twins is high (60%–90%) compared to the concordance rate among dizygotic twins (0%–30%) [10,11]. Technological advances in epidemiological and molecular genetics have led recently to new findings in the field of the genetics of neuropsychiatric disorders. These new findings concern also the domain of the genetics of autism and have increased the state of current knowledge on the genetic disorders associated with ASD. Identified genetic causes of ASD can be classified as the cytogenetically visible chromosomal abnormalities, copy number variants (CNVs) (i.e., variations in the number of copies of one segment of DNA, including deletions and duplications), and single-gene disorders [12]. CNVs can have different sizes (small to large deletions or duplications) and therefore concern a variable number of genes according to their size. The number of known genetic disorders associated with ASD has increased with the use of array Comparative Genomic Hybridization (aCGH), also called chromosomal microarrays (CMAs) or high-resolution molecular karyotype, which is one of the most molecular cytogenetic methods used by geneticists. The accuracy of aCGH is 10 to 100 times higher than low-resolution karyotype accuracy and allows detection of small CNVs. Unfortunately, this method does not detect certain genetic anomalies such as balanced chromosome rearrangements (which are rarely involved in developmental disorders) or CNVs present in less than 10% to 20% of cells (somatic mosaicism). CNVs are usually considered to be rare variants but recent techniques have shown that they may be in fact more frequent than expected [13]. In practice, after CNV detection, the geneticist needs to determine the contribution of this variation to the patient’s disorder. Most of the time, parental CNVs are also analyzed for family segregation. Among genetic causes of ASD, besides single specific genetic factors, cumulative effects of Single Nucleotide Variants (SNVs: common small variations in the DNA sequence occurring within a population) also have to be taken into consideration. Single-nucleotide polymorphism (SNP) is the term usually used for SNVs occurring in more than 1% of the general population. In the next few years, it will be probably possible to improve the identification and interpretation of SNPs in ASD using new techniques of High Throughput Sequencing, such as targeted sequencing on known genes (gene panel) or Whole Exome/Genome Sequencing. Finally, it should be highlighted that a genetic model of ASD (including cumulative genetic variance) does not exclude the role of environment (including cumulative environmental variance). It is noteworthy that heritability (*h*^2^) is defined as *h*^2^ = GV/(GV + EV) where GV is the cumulative genetic variance and EV, the environmental variance [14].

Many susceptibility genes and cytogenetic abnormalities have been reported in ASD and concern almost every chromosome (for a review, see Miles, 2011; available online: http://projects.tcag.ca/autism/) [15]. How can we explain such genetic diversity associated with similar autism cognitive-behavioral phenotypes? Given the current state of knowledge, we can state the hypothesis that the majority of ASD-related genes are involved in brain development and functioning (such as synapse formation and functioning, brain metabolism, chromatin remodeling) [16].

Several studies have reported associations between genetic contribution (such as unbalanced chromosome abnormalities, in particular large chromosomal abnormalities), dysmorphic features and low cognitive functioning [17,18,19]. Indeed, large chromosomal abnormalities are more often found in children with dysmorphic features, whereas small CNVs and de novo SNPs are more often found in individuals without dysmorphic features. The concept of “syndromic autism” or “complex autism” (autism associated with genetic disorders/genetic syndromes) which qualifies individuals with at least one dysmorphic feature/malformation or severe intellectual disability, is opposed to the concept of “non-syndromic autism” or “simplex”/“pure”/idiopatic autism (isolated autism) which qualifies individuals with moderate intellectual disability to normal cognitive functioning and no other associated signs or symptoms (except the presence of seizures) [19,20]. The prevalence of epilepsy in ASD varies from 5% to 40% (compared to 0.5% to 1% in the general population) according to different variables, including syndromic vs. non-syndromic autism (higher rates of epilepsy are observed in syndromic autism compared to non-syndromic autism) [21,22,23,24] and the age of the population (the rate of seizures varies with age and increases especially during the prepubertal period). It is noteworthy that genome-wide association and genetic analyses such as aCGH have revealed hundreds of previously unknown rare mutations and CNVs linked to non-syndromic autism. Some authors [12,19] suggest that syndromic autism (SA), compared to non-syndromic autism (NSA), is associated with a poorer prognosis, a lower male-to-female sex ratio (SA: 3/1; NSA: 6/1), and a lower sibling recurrence risk (SA: 4%–6%; NSA: up to 35%) as well as a lower risk for family history of autism (SA: up to 9%; NSA: up to 20%) given that SA would be more related to accidental mutations than NSA. Similarly, the concept of syndromic intellectual disability (intellectual disability associated with dysmorphism and medical or behavioral signs and symptoms) is widely used as opposed to non-syndromic intellectual disability (intellectual disability without other abnormalities). Intellectual disability and autism spectrum disorder share other similarities given that they are both genetically heterogeneous, and a significant number of genes have been associated with both conditions. It is noteworthy that the concept of non-syndromic autism or non-syndromic intellectual disability depends on technological progress and current state of knowledge. We can foresee, as underlined by Tordjman et al. [25], that individuals with currently and apparently “non-syndromic autism” could become a few years later individuals with “syndromic autism”, if new genetic disorders are identified, allowing a better understanding and more efficient identification of discrete clinical symptoms as minor physical anomalies, malformations and associated biological anomalies. For example, individuals with Smith–Magenis syndrome can show in early childhood ASD associated with subtle minor facial dysmorphic features (these discrete dysmorphic features become more evident later), mild or moderate intellectual disability, disrupted sleep patterns and repetitive self-hugging characteristic of this syndrome. The molecular discovery of Smith–Magenis syndrome is relatively recent (1982) and allowed to better know the characteristic behavioral phenotype combined with physical anomalies of Smith–Magenis syndrome (including short stature and scoliosis, not enough specific to help alone to identify the genetic syndrome), orienting towards the search for certain dysmorphic features and the possible identification of this genetic disorder. Thus, some children with Smith–Magenis syndrome could be considered in early childhood, especially before the discovery of Smith–Magenis syndrome in 1982, as individuals with apparently “non-syndromic autism” (they are more viewed as individuals with syndromic autism over their developmental trajectory). Therefore, taking into account this bias, it would be better to consider autism as a behavioral syndrome related either to known genetic disorders or to currently unknown causes.

The objective of this article is to show the diversity of the genetic disorders associated with ASD (including single-gene disorders, CNVs and other chromosomal disorders), and consequently to propose a hierarchical diagnostic strategy with a stepwise evaluation, helping general practitioners/pediatricians and child psychiatrists to collaborate with geneticists and neuropediatricians, in order to search for genetic disorders associated with ASD.

## 2. Known Genetic Syndromes Associated with Autism Spectrum Disorder

We chose to limit the review of literature to genetic disorders presented in Table 1. Table 1 includes one part on chromosomal disorders and another part on single-gene disorders. The present descriptions focus on autistic traits encountered in each genetic disorder and other symptoms that may be helpful for the diagnosis. Table 1 summarizes these clinical data and indicates the estimated frequency of each genetic disorder in autism and the frequency of autistic traits in each genetic disorder (when available). This table does not present an exhaustive list of genetic disorders associated with ASD. Furthermore, Table 1 includes only genetic syndromes involving a single-gene disorder or a chromosomal rearrangement, and therefore does not include polygenic causes and possible genetic disorders only related to epigenetic mechanisms. It is noteworthy that some studies suggest that the genetic background might play an important role in many ASD individuals, with in particular cumulative genetic effects related to the load of common risk variants [16].

Interestingly, as underlined by Abrahams and Geschwind [7], the descriptive analysis of Table 1 indicates that each of the many known genes or genomic regions associated with ASD, taken separately, accounts usually and on average for less than 2% of autism cases, suggesting high genetic heterogeneity (the maximum average rate is 5% and concerns the Fragile X syndrome). However, taken all together, these single genetic impairments account at least for 10%–25% of ASD individuals [244] and even 35%–40% in more recent studies [245].

## 3. Recommended Investigations for the Identification of Genetic Disorders Associated with ASD

**Figure 1:**
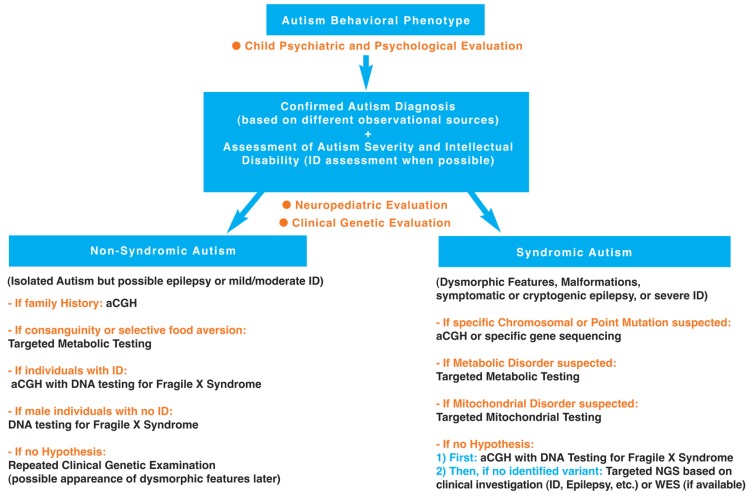
Hierarchical diagnostic strategy with a stepwise evaluation towards identification of genetic disorders associated with autism.

Facing such a diversity of genetic disorders associated with ASD (see Table 1), it seems important to clarify, as best as possible with regard to the current state of knowledge, the diagnostic steps towards identification of these genetic disorders. A hierarchical diagnostic strategy with a stepwise evaluation towards identification of genetic disorders associated with autism is proposed and developed in Figure 1.

As it appears in Table 1, the severity of the autistic syndrome is an important variable to take into consideration in the search for genetic disorders associated with ASD. Therefore, the preliminary step in the hierarchical diagnostic strategy is to confirm autism diagnosis and assess autism severity. Furthermore, based on the genetic syndromes listed in Table 1, certain variables are of particular interest such as dysmorphic features, malformations, epilepsy and intellectual disability. It indicates clearly that clinical investigation of behavioral phenotype of autism for identification of genetic disorders associated with ASD requires clinical genetic examination, neuropediatric examination and psychological assessment. In this section, we propose a hierarchal diagnostic strategy with focused investigations for the identification of genetic disorders associated with ASD, based on the history of the individual (including family history and developmental trajectory) and the clinical neurologic and genetic evaluation, but also on up-to-date knowledge and new technology. The first step of the hierarchal diagnostic strategy is a clinical investigation involving: (i) a child psychiatrist’s and psychologist’s evaluation to confirm autism diagnosis using different observational sources (including an extensive parental interview facilitating the study of family history) and assess autism severity (including behavioral and cognitive assessments); (ii) a neuropediatrician’s evaluation to examine neurological symptoms/signs and developmental milestones; and (iii) a geneticist’s evaluation to search for dysmorphic features and malformations based on a clinical physical examination. This clinical step is followed by a second step involving laboratory and if necessary neuroimaging or EEG studies oriented by clinical results based on clinical genetic and neuropediatric examinations. These two steps are described below.

### 3.1. First Step: Clinical Investigation

#### 3.1.1. Autism Diagnosis Using Different Observational Sources and Autism Severity Assessment (Child Psychiatric and Psychological Evaluation)

The preliminary step requires confirming the diagnosis of autism and assessing the severity of autistic behavioral impairments with validated tools used by trained professionals in different observational situations, such as the Autism Diagnostic Interview-Revised [246] (the ADI-R scale is a parental interview) and the Autism Diagnostic Observation Schedule [247] (the ADOS scale allows a direct observation of the individual through a standardized play situation). The psychiatric appreciation of ICD-10 (International Classification of Diseases, World Health Organization, Geneve, Switzerland, 1993) and DSM-5 (American Psychiatric Association, Washington, D.C., United States, 2013) diagnostic criteria of autism is also necessary and provides a clinical psychiatric judgment. This approach, combining information from multiple sources based on the clinical psychiatric judgment and the administration of the ADI-R completed by the ADOS improves the confidence in the diagnosis of ASD [248]. Interestingly, the ADI-R scale is validated to assess current behavior but also behavior during the 4–5 years old period (the ADI-R algorithm is based on the 4–5 years old period of life which is supposed to correspond to the most severe period of autistic impairments), allowing to see the evolution between these two periods of life. It happens more frequently than expected that children who fulfill the diagnostic criteria for ASD based on the ADI-R algorithm (parental interview for the 4–5 years old period) do not meet the full diagnostic criteria for ASD based on the ADOS algorithm (direct observation of the child for the current period).

The ADI-R scale is the most common assessment used to conduct an extensive semi-structured parental interview searching for medical and psychiatric family history. This clinical interview should be conducted with empathy and allow a three-generation family tree to be constructed, showing, in particular, family psychiatric history (such as, for example, schizophrenia, depression or bipolar disorder) and ASD behaviors (including delayed language development) within the family (mother’s and father’s sides). The construction of the pedigree can be very helpful to get specific hypotheses of genetic disorders associated with autism. More generally, higher rates of CNVs are observed in ASD individuals with a family history of psychiatric disorders or developmental disabilities, highlighting the importance to study family history [249]. It is noteworthy that family history is also investigated and analyzed by the neuropediatrician and the geneticist during their evaluation.

Furthermore, the clinician should indicate in the family tree the existence of consanguineous mating, family malformations, deceased infants and spontaneous abortions occurring in the mother but also in relatives (repeated spontaneous abortions are in favor of chromosomal rearrangement [20,250,251]). It is necessary to specify the trimester of pregnancy when spontaneous abortions occurred given that repeated miscarriages at the first trimester of pregnancy are more specifically in favor of genetic anomalies. Reproductive stoppage is rarely evoked but should be investigated because reproductive curtailment following the birth of a child with ASD has been reported [252]. In addition, the birth order of the child with autism has to be noted in the family tree given that a decrease of ASD in later siblings has been observed [253]. The maternal and paternal ages at the time of procreation should be also systematically noted. Indeed, several studies suggest that advanced parental age, in particular for the father, is a risk factor for ASD [254], but there are some discrepancies in the results [253]. Although advanced parental age may be a risk factor, it orients towards genetic testing but does not lead to definitive conclusions.

Finally, a psychological evaluation is necessary in this hierarchical diagnostic strategy to assess the level of cognitive functioning given that intellectual disability and its severity are associated, as indicated previously, with a risk for several genetic disorders in ASD individuals (see Table 1). In addition, the level of cognitive functioning should be taken into consideration and therefore assessed given that severe intellectual disability can introduce an important bias leading to a misdiagnosis of autism. Indeed, ASD is often misdiagnosed in individuals with severe intellectual disability. It should be noted that the diagnosis of autism might be not valid or relevant for genetic disorders associated with severe intellectual disability due to first, the behavioral overlap between severe intellectual disability and autistic behavioral impairments (social communication deficit as well as repeated behaviors or interests), and second, the absence of validity and reliability of autism diagnostic instruments in the context of very low IQ or mental age of less than 24 months [255]. It is noteworthy that the most currently international instruments used for autism diagnosis, such as the ADI-R and the ADOS scales, were not normed in individuals with severe intellectual disability [255]. Cognitive functioning can be assessed, whenever possible with the child, by a psychologist or a neuropsychologist if available, using the age-appropriate Wechsler intelligence scales (Wechsler Preschool and Primary Scale of Intelligence: WPPSI-IV for children between 2 and 7 years old, Wechsler Intelligence Scale for Children: WISC-5 for children between 6 and 16 years old, Wechsler Adult Intelligence Scale: WAIS-IV for individuals above 16 years old) or the Kaufman Assessment Battery for Children (K-ABC ) [256]. The Kaufman K-ABC is supposed to be more adapted for nonverbal children but cognitive assessment appears in fact easier to perform using the Wechsler intelligence scales with nonverbal subtests for nonverbal ASD children. Some instruments are of particular interest for intellectual disabled children with ASD such as the Raven’s Color Progressive Matrices (CPM) which is a short (20 min) nonverbal intelligence test validated for young children and individuals with intellectual disability [257].

The child psychiatric evaluation and psychological evaluation confirming autism diagnosis from different observational sources and assessing autism severity and intellectual disability are followed by a neuropediatrician’s evaluation and a geneticist’s evaluation (see Figure 1).

#### 3.1.2. Developmental Milestones (Neuropediatric Evaluation)

A neuropediatric examination should be systematically conducted searching for neurological symptoms associated with ASD such as hypotonia, ataxia, abnormal movements or epilepsy. In case of clinical symptoms of epilepsy, the electroencephalogram (EEG) can be a useful tool to detect epileptic brain activity. In complement to this neuropediatric examination, it is necessary to rule out a sensory deficit, such as an auditory or visual deficit, based on ophtamological and ears-nose-throat examination (an audiogram is recommended). In addition, the neuropediatric examination investigates extensively developmental milestones for the following periods (some developmental milestones are often explored also during the child psychiatric evaluation): (i) prenatal period: course of pregnancy, fetal movements, results of ultrasound exams, treatments (in particular, in-utero exposure to thalidomide or valproate, known as teratogenic medications and risk factors for ASD), and other possible prenatal risk factors for ASD (such as gestational diabetes, gestational bleeding or multiple birth [6]); (ii) perinatal period: birth presentation (abnormal presentation in general and breech presentation in particular, have been associated with the development of ASD [258], mode of delivery, clinical status at birth (APGAR, score, weight, body length, and head circumference), premature birth (the risk for autism increased with the severity of preterm birth [259]), conditions related to hypoxia at birth reported as risk factors for ASD (such as umbilical cord complications, meconium aspiration, neonatal anaemia, ABO or Rh incompatibility, hyperbilirubinemia, or birth injury and trauma; for a review, see Gardener et al. [260]), feeding pattern and difficulties, screening tests (hypothyroidism, phenylketonuria), physical examination (apparent dysmorphism and malformations) and neurological examination (neonatal hypotonia); and (iii) postnatal period: psychomotor development, feeding pattern and possible gastrointestinal problems, sleep pattern, epilepsy, sensory dysfunction, language development, early behavioral signs of autism and other atypical behaviors. This clinical evaluation investigates developmental and learning disorders. The study of the height-weight-head circumference growth curve can show a break, decline with regression, or decrease with stagnation. For example, a decrease between 6 and 18 months of head circumference growth should orient the clinician towards the search for Rett syndrome in its first stage (stagnation stage) which occurs before the stage of fast decline of the communication skills (regression stage between 12 and 18 months of age). In addition, a developmental regression should orient the clinician towards the search for metabolic diseases. More generally, neuroimaging is recommended in individuals with ASD and microcephaly [249]. It is noteworthy that parental measures of height-weight-head circumference have also to be considered in order to determine whether the child’s height-weight-head circumference measures are really abnormal compared to the parents’ ones or whether a parent may be mildly affected, orienting towards a congenital or acquired abnormality.

#### 3.1.3. Physical Examination (Genetic Evaluation)

A clinical genetics evaluation is systematically required and involves an extensive physical examination of the whole body. This physical examination should be conducted by a clinical geneticist trained in dysmorphology searching for dysmorphic features or malformations, and includes photos of the head (face and profile) with a good visibility of the ears, hands (palm and dorsal view) and feet. When clinical geneticists are not easily available, alternative options are to send, as a preliminary step, patients’ pictures (face and hands pictures) to a geneticist for a specialized opinion (our group is testing the feasibility and efficiency of such a procedure and is comparing the results obtained based on the analyses of pictures by a group of geneticists to the ones obtained based on the analyses of the same pictures using the genetic search and reference mobile application Face2Gene developed by FDNA: Facial Dysmorphology Novel Analysis). In addition, the skin should be examined with a Wood lamp, in particular when tuberous sclerosis is suspected [251]. Finally, clinical genetics evaluation can greatly benefit, in this strategy of identification of genetic disorders associated with ASD, from examining repeatedly over time the child to follow his/her developmental trajectory and examining at the same time the patient, his/her parents and siblings to compare the findings.

### 3.2. Second Step: Complementary Investigations (Laboratory Studies)

This secondary investigation includes cytogenetic, molecular analyses, biochemical analyses. Other exams such as neuroimaging and EEG are requested based on the results of the clinical genetic and neuropediatric examinations.

The following hierarchical diagnostic strategy, in line with recent American guidelines [249], can be proposed (see Figure 1).

#### 3.2.1. Non-Syndromic Autism (Isolated ASD)

It should be remembered that non-syndromic autism includes ASD individuals with moderate intellectual disability to normal cognitive functioning (high functioning ASD) and no other associated signs or symptoms (except the possible presence of seizures). To our knowledge, the probability to detect a mutation (CNV or point mutation) is very low in high functioning non-syndromic ASD individuals, and, in this case, we do not recommend molecular analysis. However, in the case of family history of psychiatric disorders or developmental disabilities, it could be of interest to perform aCGH and to collect samples for a research program. In addition, in the case of consanguinity or selective food aversion, it could be useful to perform targeted metabolic testing. In addition, in the case of individuals with intellectual disability or males with no intellectual disability, the search for Fragile X mutation is recommended. If there is no hypothesis, a repeated clinical genetic evaluation over time is proposed given the possible later appearance of dysmorphic features along with the developmental trajectory.

#### 3.2.2. Syndromic Autism (ASD Associated with Dysmorphic Features and/or Malformations and/or Symptomatic or Cryptogenic Epilepsy, and/or Severe Intellectual Disability)

If a specific chromosomal or point mutation is suspected: perform aCGH or specific gene sequencing.

If a metabolic disorder is suspected based on developmental regression (regression observed on the growth curves, including macrocephaly and in particular microcephaly, but also developmental psychomotor regression or neuroregression/neurodegeneration with neurological symptoms), seizures and especially epilepsy (recurrent and refractory seizures), consanguinity and specific dysmorphy or other symptoms described below: perform targeted metabolic testing including measurements of ammonemia and lactatemia, urinary organic acids chromatography, plasmatic amino-acids chromatography, creatine metabolism, iron, vitamin D, glutathione, oxidative stress, and cerebral magnetic resonance spectroscopy combined with standard neuroimaging. A metabolic disease could also be suspected based on certain clinical symptoms, such as digestion-related symptoms (cyclic vomiting, selective eating or gastrointestinal problems), dermatologic and hair changes (rashes, pigmented skin eruptions, hypertrichosis or alopecia), lethargy with hypotonia/extrapyramidal signs (dystonia, abnormal movements), or multiple organ dysfunction (in particular, heart, liver, kidney). In addition, the search for metabolic disorders can be indicated by abnormal biological measures such as anemia or lactic acidosis, and more generally acid/base or electrolyte disturbances. Searching for metabolic disorders is important given the potential availability of treatments such as enzymotherapy for certain metabolic diseases, diet low in phenylalanine for phenylketonuria due to the enzyme phenylalanine hydroxylase deficiency, or supplementation with folinic acid for cerebral folate deficiency. Several metabolic disorders associated with ASD are listed in Table 1 using the superscript **#**.

If a mitochondrial disorder is suspected based on repeated regressions after three years old, neurological abnormalities (including hypotonia) and multiple organ dysfunction: perform targeted mitochondrial testing including measurement of lactatemia. Abnormal neurologic examination and/ or elevated plasma lactate concentration have been indeed found in mitochonfrial diseases [260]. A mitochondrial disorder could be also suspected in case of ASD associated with loss of speech after a febrile illness or immunization with encephalopathy [261]. Finally, it is noteworthy that mitochondrial disease (rare in ASD) has to be differentiated from mitochondrial dysfunction (common in ASD) [262].

If there is no hypothesis of specific syndrome: perform aCGH and molecular diagnosis of Fragile-X syndrome. Without any identified pathogenic variant, we recommend targeted New Generation Sequencing (NGS) on known genes (for example, panels of genes related to intellectual disability or epilepsy based on the clinical examination) or Whole Exome Sequencing (WES) if available.

## 4. Conclusions

The finality of the hierarchal diagnostic strategy proposed in this article is to identify genetic disorders associated with ASD based on a stepwise evaluation characterized by low invasiveness and cost but high accessibility, feasibility and adaptability of current knowledge and technology to the individual and his/her family. These clinical and laboratory investigations can lead to identify genetic disorders associated with ASD in 35%–40% of individuals [225,262]. The identification of a specific genetic syndrome associated with ASD has practical clinical implications, in terms of diagnostic strategies including early detection and prevention of co-morbidity related to the known medical risks of the genetic disorder, follow up adapted to the genetic disorder with a more precise prognosis, and therapeutic strategies with access to needed services, help provided by specific family associations or specific individualized treatment in some cases. In many cases, identification of genetic disorders does not lead to therapeutic implications but there are a few cases where enzymotherapy, food depletion or supplementation in certain metabolic diseases as well as administration of melatonin associated with acebutolol in Smith–Magenis syndrome have improved considerably the quality of life of patients and their families. It should be noted that sleep problems are frequent in ASD (prevalence of insomnia: 50%–80% [263]) and decreased nocturnal and/or diurnal levels of melatonin (a sleep neurohormone) have been reported in many ASD studies; trial studies support therapeutic benefits of melatonin use in ASD (for a review, see Tordjman et al. [116,264]. Furthermore, the identification of genetic disorders associated with ASD allows unique personalized genetic counseling adapted to each family with regard to the transmission risk to the descendants and the possibility of a prenatal or preimplant diagnosis in the perspective of a future pregnancy. According to the 2013 American guidelines [249], the genetic counselor should note the type of array technology used (e.g., oligonucleotide vs. single-nucleotide polymorphism arrays) and access major databases relevant to this technology in order to provide the best possible and adapted information for the family. Many parents of children with ASD worry about the autism risk for a future pregnancy and knowing that their impaired child has a de novo mutation can be very helpful concerning the decision to have another child. It can be noted that the majority of the found mutations or rearrangements occurs de novo and are therefore not inherited from the parents or relatives [262]. In the same vein, siblings of children with ASD are very often preoccupied by the possible genetic transmission of autism to their future children. This preoccupation is legitimate considering, for instance, that the sisters of a boy with Fragile X Syndrome, even if they express very few or no symptoms, have a risk of 50% to carry the mutation or premutation and therefore a risk of 50% to have a child with Fragile X Syndrome (more severely impaired if it is a male). It is noteworthy that genetic counseling is also offered to families of ASD individuals without identified etiology, using in this case epidemiologic studies, and in particular sibling recurrence-risk updated data [249]. In any case, genetic counseling provides helpful family guidance, and parents of children with ASD are often relieved that biological factors involved in the development of ASD are considered and possibly found when some of them might feel guilty with regard to the role of family environment in ASD. Interestingly, based on our experience and the experience of geneticists or neuropediatricians working with psychoanalysts [265], knowing the underlying genetic cause of a relative’s or patient’s disorder, far from fixing the family or caregiver representations with regard to genetic determinism, rather creates and stimulates new dynamics within the family as well as the caregiver team centered on the ASD individual and probably related to a better known developmental trajectory with therapeutic perspectives and a parental relief. Finally, given all these implications, it is essential that all individuals (children but also adolescents and adults) with ASD can benefit from a clinical genetic examination and neuropediatric examination searching for associated signs or symptoms helping to identify known genetic disorders.

## Figures and Tables

**Table 1:** Main genetic disorders associated with autistic syndrome.

Genetic Disorder [References]	Estimated Rate (%) of the Disorder in Autism	Estimated Rate (%) of Autism in the Disorder	Degree of Intellectual Disability (ID)	Possible Autistic Behaviors	Other Possible Behaviors	Other Possible Symptoms
**Chromosomal Disorders**						
Maternal * 15q11–q13 duplication [26,27,28,29,30,31,32,33,34,35,36,37,38]	1–2	80–100	Severe	Severe autistic syndrome with severe expressive language impairment	Hyperactivity and aggression	Seizures (75%), hypotonia, genitor/urinary abnormalities
Angelman syndrome * (maternal 15q11-q13 deletion, paternal uniparental disomy, mutations of *UBE3A* that encodes an ubiquitin E3 ligase) [35,39,40,41,42,43,44,45,46,47,48]	1	48–80	Severe	No language, stereotypies, sameness	Attention Deficit with Hyperactivity Disorder (ADHD), paroxysmal laughter, tantrums	Facial dysmorphism, microcephaly, seizures (>1 year), ataxia and walking disturbance
Prader–Willi syndrome * (maternal uniparental disomy at 15q11-q13, paternal deletions) [35,49,50,51,52,53,54,55,56,57,58] Paternal 15q11-q13 duplication (only few cases reported to be associated with autism): behavioral phenotype similar to Prader–Willi syndrome [58,59,60]	Not Available (NA)	19–37	Mild to moderate	Motor and verbal stereotypies, rituals	Hyperphagia, obsessive-compulsive traits, temper tantrums	Obesity, growth delay and hypogonadism, facial dysmorphism, Hypotonia
Phelan–McDermid * syndrome (Inherited, de novo deletions at 22q13.3 leading to loss of *SHANK 3*) 22q13.3 duplication [20,61,62,63,64,65,66,67,68,69,70]	NA	75–84	Severe	Variable autistic syndrome with social communication impairments, including delayed or absent verbal language	Global developmental delay, atatonia in adolescence and adulthood	Dysmorphic features, hypotonia, gait disturbance, recurring upper respiratory tract infections, gastroesophageal reflux and seizures
16p11.2 duplication 16p11.2 deletion [71,72,73,74,75,76,77]	1	33	Severe	Severe autistic syndrome with speech impairment	Gross and fine coordination problems, SCH (Schizophrenia), anxiety, ADHD	Hypotonia (Multiple congenital anomalies are possible with more distal region)
Inverted duplication/deletion 8p21–23 [78,79]	NA	30–57	Variable	Mild to moderate autistic syndrome with absent or delayed verbal language	ADHD	Minor facial dysmorphism, hypotonia, agenesis of the corpus callosum, possible heart defect
Genetic disorder [References]	Estimated rate (%) of the disorder in autism	Estimated rate (%) of autism in the disorder	Degree of intellectual disability (ID)	Autistic behaviors	Other behaviors	Other symptoms
Down syndrome * (trisomy 21) [80,81,82,83,84]	2	5–10	Variable but usually severe when autism	Severe autistic syndrome	-	Facial dysmorphism, heart and intestine malformations
Smith–Magenis syndrome (17p11.2 deletion) [85,86,87,88]	<1	80–100	Variable	Self-injurious and stereotyped behaviors, sameness	Tantrums, possible social contact, sleep disturbance	Facial dysmorphism, peripheral neuropathy, hypotonia
Potocki–Lupski syndrome (17p11.2 duplication) [89]	NA	50–100	Normal to moderate	Decreased eye contact, motor manierisms or posturing, sensory hypersensitivity or preoccupation, repetitive behaviors or interests, lack of appropriate functional or symbolic play and lack of joint attention	Developmental delay, language impairment, and cognitive impairment	hypotonia, poor feeding and failure to thrive in infancy, oral-pharyngeal dysphagia, obstructive and central sleep apnea, structural cardiovascular abnormalities, electroencephalography (EEG), abnormalities, and hypermetropia
2q37 deletion [90,91,92,93,94]	<1	25–35	Mild to moderate	Severe communication impairment, stereotypies	Hypotonia, hyperactivity, Obsessive-Compulsive Disorder (OCD), aggression, sleep disturbance	Facial dysmorphism, microcephaly, growth delay/short stature, intestine and heart malformations, seizure
22q11.21 duplication and 22q11 deletion (DiGeorge/Vélocardio-facial syndrome) [95,96,97,98,99,100]	NA	<10	Normal to severe ID	Autistic syndrome, Pervasive Developmental Disorder-Not Otherwise Specified PDD-NOS (ICD-10 criteria)	Learning disability, anxiety, ADHD, oppositional-defiant disorder, OCD, motor impairment	Facial dysmorphism, microcephaly, growth delay/short stature, craniofacial abnormalities/cleft palate, heart defect, hypotonia
1q21.1 Copy-Number Variation (CNV) (1q21.1 deletion/duplication) [101,102,103,104]	NA	<30	Normal to mild ID	Autistic syndrome, PDD-NOS (ICD-10 criteria)	Developmental delay, learning disability, anxiety, ADHD, aggression, SCZ and hallucination	Microcephaly (deletion) Macrocephaly (duplication)
Williams-Beuren syndrome * (7q11.23 deletion) and Reciprocal 7q11.23 duplication syndrome [105,106,107,108,109,110,111,112,113,114,115,116,117]	<1	<10	Mild to moderate	Autistic syndrome	Overfriendliness, over talkativeness, visual spatial deficit, hyperacusis, feeding and sleep problems	Facial dysmorphism, short stature, heart and endocrine malformations, hypercalcemia
Turner syndrome * (most common monosomy For X chromosome) [37,118,119]	NA	3	Usually normal IQ	Females monosomic for the maternal chromosome X score significantly worse on social adjustment and verbal skills	-	Short stature, skeletal abnormalities, absence of ovarian function, webbed neck, lymphedema in hands and feet, heart defects and kidney problems
Beckwith–Wiedemann * syndrome (abnormal expression of imprinted genes on chromosome 11p15.5 such as *IGF2* and/or *CDKN1C*) [120,121,122,123]	NA	6.8 (replication needed)	Usually normal IQ	Autistic syndrome	-	Pre- and postnatal overgrowth (hemihyperplasia, macroglossia, visceromegaly) and increased risk of embryonal tumors
Isodicentric chromosome 15 or duplication/inversion 15q11 [124,125]	NA	NA	Moderate to severe	Autistic behavior	Developmental delay and intellectual deficit, epilepsy	Early central hypotonia
Ito hypomelanosis [126,127]	NA	NA	Inconstant	Asperger syndrome(high functioning autism) or atypical autism	Psychomotor delay and cognitive deficit	hypopigmented skin lesions along the Blaschko lines, motor delay, seizures, microcephaly or macrocephaly, hypotonia, ophthalmological abnormalities
**Single Gene Disorders**						
CHARGE syndrome * (*CHD7*, 8q21.1) [128,129,130,131,132,133]	<1	15–50	Variable but often normal IQ	Variable autistic syndrome	Hyperactivity, obsessive-compulsive traits, tic disorders	Coloboma of the eye, Heart defects, Atresia of the nasal choane, Retardation of growth and/or development, Genital/urinary abnormalities, Ear abnormalities/deafness
Tuberous sclerosis (*TSC1*, 9q34) (*TSC2*, 16p13.3) [134,135,136]	1–4	25–60	Variable	Severe autistic syndrome	Learning disorder	Ectodermal anomalies, renal lesions, seizures
PTEN macrocephaly syndromes (*PTEN*, 10q23.31) [137,138,139,140,141,142]	4 in ASD with macro-cephaly	NA	Severe	Autistic syndrome and language delay	=	Progressive macrocephaly, developmental delay, macrosomy, tumors in adulthood
Rett’s syndrome * (*MECP2*, Xq28) [117,143,144,145,146,147,148,149]	<1 in female	61–100	Severe	Stereotyped hand movements, absence of language, loss of social engagement	Stagnation stage (6–18 months) in girls, then regression stage (12–36 months), pseudostationary t stage (2–10 years), l and late motor t deterioration (>10 years)	Head growth deceleration, progressive motor neuron (gait and truncal apraxia, ataxia, decreasing mobility) and respiratory (hyperventilation, breath holding, apnea) symptoms
San Filippo syndrome ^#^ (*SGSH*, 17q25.3) [150,151,152,153]	1 replication needed	80–100	Severe	Language impairment, autistic withdrawal, stereotyped behaviors impulsivity, inappropriate affects	Progressive loss of acquisitions	Motor regression, hepatomegaly
Cerebral folate deficiency ^#^ (*FOLR1*, 11q13.4) [154,155,156,157]	NA	NA	Variable	Autistic syndrome including especially social interaction and language impairment	irritability, movement (such as tremors) and gait disturbances with ataxia, sleep problems	Psychomotor regression, epilepsy, developmental delay, deceleration of head growth, dystonia/hypotonia, visual and hearing deficit
Smith–Lemli–Opitz syndrome ^#^ (*DHCR7*, 1q12–13) [158,159,160,161,162,163]	NA	50	Variable	Self-injurious behaviors, stereotypies (“opisthokinesis”) language impairment	Sensory hyper-reactivity, irritability, sleep disturbance	Facial dysmorphism, cleft palate, congenital heart disease, hypospadias, 2–3 toe syndactyly
Phenylketonuria ^#^ (*PAH*, 12q22-q24.1) [20,164,165]	NA	NA	Severe	Self-injurious behavior, lack of social responsiveness	Temper tantrums, hyperactivity	Eczema, hypertonia, seizures, hypo-pigmentation
Adenylosuccinate lyase deficiency ^#^ (*ASL*, 22q13.1–13.2) [166,167,168,169,170]	<1	80–100	Variable	Severe autistic syndrome	-	Seizures
Creatine deficiency syndrome ^#^ (*GAMT*, 19p13.3) (*CRTR*, Xq28) [171,172,173,174]	<1	80–100	Severe	Severe autistic syndrome with poor language	-	Seizures, hypotonia
*SHANK* *3* (22q13.3) [175,176,177]	<1	NA	NA	Severe autistic syndrome with no language	-	-
Neurexin family: Neurexin 1 (*NRX1*, 2p16.3) [178,179,180,181,182]	1	NA	Variable	Autistic syndrome	Hyperactivity, depression, learning disability, but also normal behavior	Seizures, hypotonia, facial dysmorphism?
Contactin Associated Protein-like 2 (*CNTNAP2*, 7q35) [183,184,185]	NA	NA	Variable	Autistic syndrome including verbal language impairment	-	Seizures
Contactin 4 (*CNTN4*, 3p26.2–3p26.3) [186,187,188,189]	<1	NA	Variable	Autistic syndrome, PDD-NOS (ICD-10 criteria)	Visual spatial impairment, regression	Facial dysmorphism, developmental delay, hypotonia, ptosis.
Cell adhesion molecule-1 (*CADM1*, 11q22.3–23.2) [190,191,192]	NA	NA	NA	Autistic syndrome with especially social communication impairment including verbal language deficit	-	-
Protocadherin 10 (*PCDH10*, 4q28) [193]	<1	NA	NA	Autistic syndrome	-	-
Neuroligin family: Neuroligin 3 (*NLG3*, Xq13) Neuroligin 4 (*NLG4*, Xq22.33) [194,195,196]	<1	NA	Variable	Severe autistic syndrome, PDD-NOS (ICD-10 criteria)	Regression	Tic
Fragile X * (*FMR1*, Xq27.3) [191,196,197,198,199,200,201,202,203,204,205,206,207,208,209,210,211,212,213,214,215,216,217,218,219,220,221,222]	0–8	0–33	Variable	Poor eye contact , social anxiety, language deficit and stereotypies	Hyperactivity with attention deficit, sensory hyper-reactivity	Facial dysmorphism, macro-orchidism
Neurofibromatosis type 1 [109,223,224,225]	NA	21–40	inconstant	Restrictive/repetitive behaviors and severe social-communicative impairments	Cognitive deficits and learning difficulties	Café-au-lait spots, iris Lisch nodules, axillary and inguinal freckling, multiple neurofibromas
Sotos syndrome [226,227]	NA	70 (replication needed)	Mild to severe	Self-injurious behavior, physical aggression, and destruction	-	Facial dysmorphism, overgrowth of the body in early life with macrocephaly
Aarskog syndrome [228,229]	NA	NA	-	Variable autistic syndrome	Learning and behavioral disabilities	facial, limbs and genital features, acromelic short stature
Cornelia de Lange syndrome [117,230,231]	NA	43 (replication needed)	Variable	Self-injury, excessive repetitive behaviors and expressive language deficits	Psychomotor delay, language acquisition difficulties	Facial dysmorphism, severe growth delay, abnormal hands and feet, and constant brachymetacarpia of the first metacarpus, various other malformations (heart, kidney, etc.)
Joubert syndrome [232]	NA	<40	severe intellectual deficit to normal intelligence	Autistic syndrome	-	Facial dysmorphism, abnormal respiratory pattern, nystagmus, hypotonia, ataxia, and delay in achieving motor milestones
Cohen syndrome [117,233,234,235]	NA	54 (replication needed)	variable	Atypical autism	Often sociable with a cheerful disposition, learning and behavioral problems	Microcephaly, facial dysmorphism, hypotonia, myopia, retinal dystrophy, neutropenia and truncal obesity
Lujan–Fryns syndrome [236]	NA	NA	Mild to moderate	Autistic-like disorder	Behavioral problems, emotional instability, hyperactivity and shyness, schizophrenia	Tall, marfanoid stature, facial dysmorphism, hypernasal voice and generalized hypotonia
Noonan syndrome [237,238,239]	NA	15 (replication needed)	Mild	ASD	Poor feeding in infancy	Short stature, facial dysmorphism and congenital heart defects
Moebius syndrome [128,240,241]	NA	0–45	Inconstant, mild	ASD	Delayed walk development	Oculofacial paralysis, strabismus
Helsmoortel–Van der Aa syndrome (ADNP-related ID/ASD) [242]	0.17	100	Mild to severe	ASD, behavioral problems, sleep disturbance	Delayed developmental milestones (walking between 19 months and 4.5 years, and speech ranging from sentences to no words)	Facial dysmorphism, hypotonia, seizures, feeding difficulties, visual problems (hypermetropia, strabismus, cortical visual impairment), and cardiac defects
Timothy syndrome (CACNA1C) [243]	NA	50–70 (replication needed)	Inconstant, mild to severe	ASD	Language, motor, and generalized cognitive impairment	Facial dysmorphism, rate-corrected QT (QTc) interval >480 ms, unilateral or bilateral cutaneous syndactyly, heart defects, seizures

Adapted from Tordjman et al. [25]. ^#^ These genetic disorders are metabolic disorders (the list is not exhaustive). * The asterisk indicates the existence of epigenetic mechanisms observed in these genetic disorders (the list is not exhaustive).
